# Supplementary material for: High-throughput production of functional prototissues capable of producing NO for vasodilation
Source: Nat Commun. 2022 Apr 20;13:2148. doi: 10.1038/s41467-022-29571-3 (PMC9021269; doi:10.1038/s41467-022-29571-3)
Supplement: Supplementary file 1 — Supplementary Information [file 41467_2022_29571_MOESM1_ESM.pdf]

Supplementary Information

High-throughput production of functional prototissues capable of producing NO for vasodilation

Han et al.

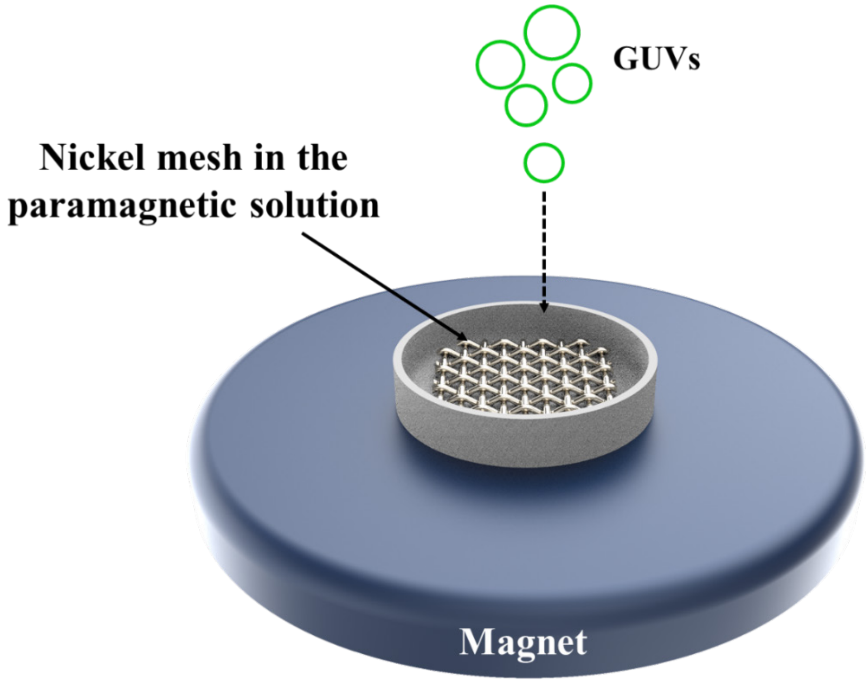

Supplementary Fig. 1 Schematic illustration of home-made device for prototissue formation.

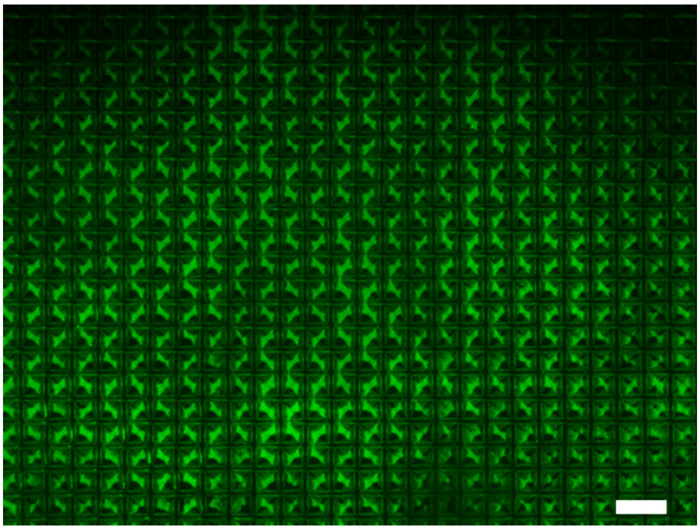

Supplementary Fig. 2 Fluorescence zoom out image (from at least 3 independent samples) of the GUVs prototissue array. The scale bar was 400 μm.

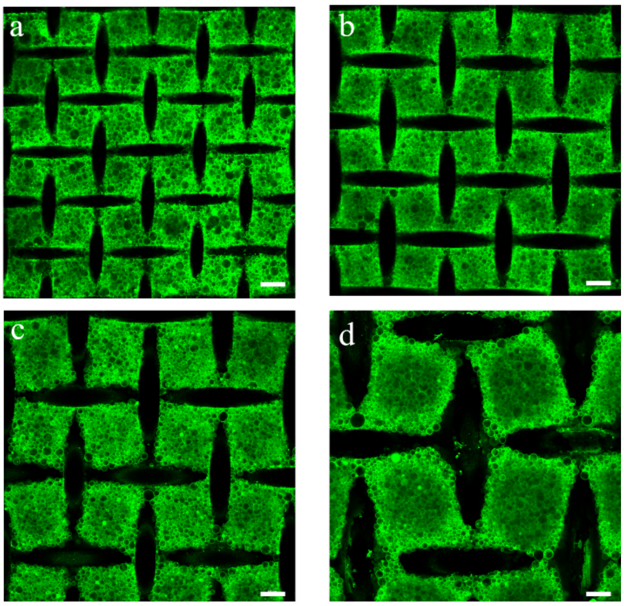

Supplementary Fig. 3 GUVs prototissues assembled using the NMs with different side length of 150 μm **(a)**, 180 μm **(b)**, 230 μm **(c)**, and 350 μm **(d)**. The representative fluorescence images from at least 3 independent samples. The scale bars were 100 μm.

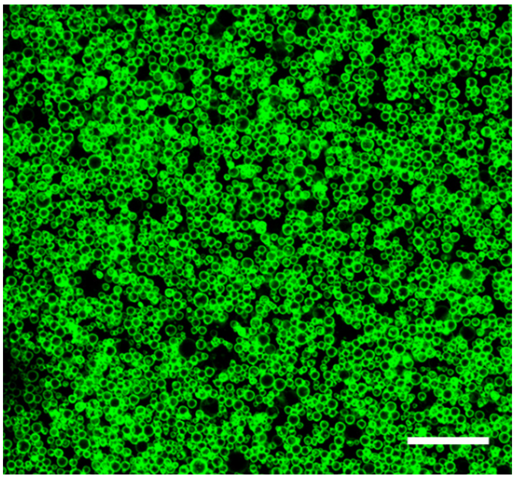

Supplementary Fig. 4 A fluorescence image (from 5 independent samples) of the distribution of GUVs with no magnetic field. Random GUVs distribution was observed. The scale bar was 100 μm.

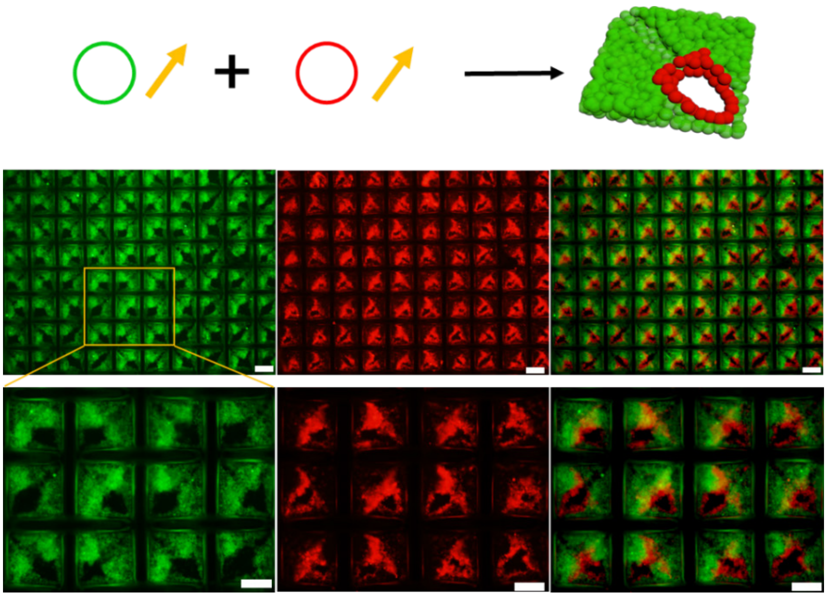

Supplementary Fig. 5 Schematic and fluorescence images (from 3 independent samples) of the two-component GUVs prototissues. Yellow arrow indicated the inclined magnetic field. Red and green rings indicated the red and green GUVs, respectively. The prototissues inside each grid were assembled by successively trapping gGUVs (3×10^5/mL) and rGUVs (1.5×10^5/mL) under inclined magnetic field. The images in the bottom row were partial enlargements of the images in the top row. The left and middle column images were the prototissues viewed by green channel and red channel, respectively. The right column images were the merged images of left and middle images. The scale bars were 100 μm.

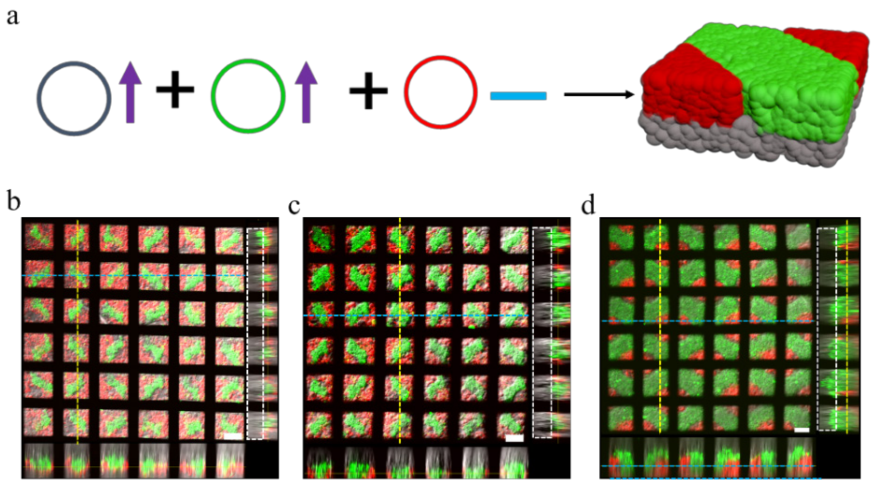

Supplementary Fig. 6 Three-component GUVs prototissues. **(a)** Schematic of the three-component prototissues assembled by successively trapping non-labeled GUVs and gGUVs under vertical magnetic field, and rGUVs with no magnetic field. Purple arrow and cyan line indicated the vertical and no magnetic field, respectively. Red, green and gray rings indicated the red, green and non-labeled GUVs, respectively. **(b), (c), (d)** Fluorescence images (from 3

independent samples for each case) of the three-component GUVs prototissues with varying rGUVs and gGUVs. The concentrations of the non-labeled GUVs were all 6×10^5/mL in **b, c, d**. The concentrations of the gGUVs were 3×10^5/mL, 4×10^5/mL and 6×10^5/mL in **b, c, d**, respectively. The concentrations of the rGUVs were 5×10^5/mL, 4×10^5/mL and 2×10^5/mL in **b, c, d**, respectively. The non-labeled GUVs populations were indicated by the white dashed boxes. The scale bars were 100 μm.

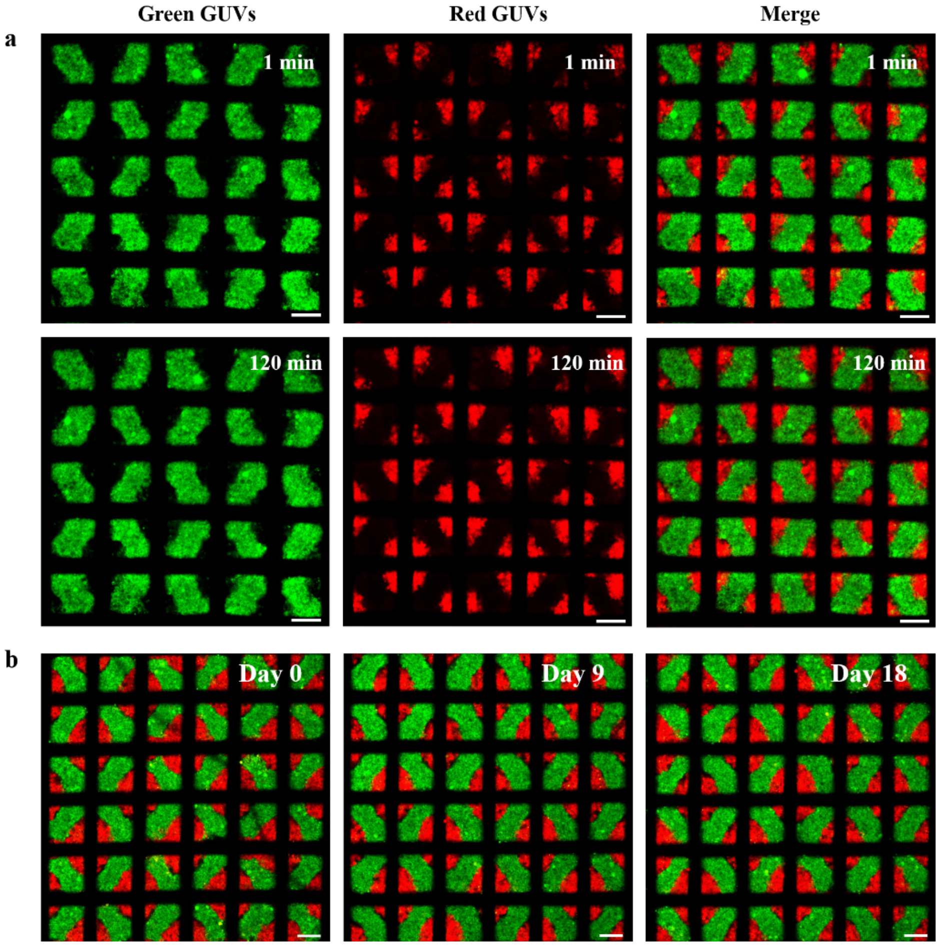

Supplementary Fig. 7 Stability of the three-component prototissues. **(a)** Fluorescence images (from 3 independent samples) of the prototissues on the nickel mesh being inverted for 1 min (top row) and 120 mins (bottom row) respectively. Green GUVs populations assembled with magnetic field (left column), red GUVs populations assembled without magnetic field (middle column) and their merged images (right column). **(b)** Fluorescence images (from 3 independent samples) of prototissues on the nickel mesh at different days. The scale bars were 100 μm.

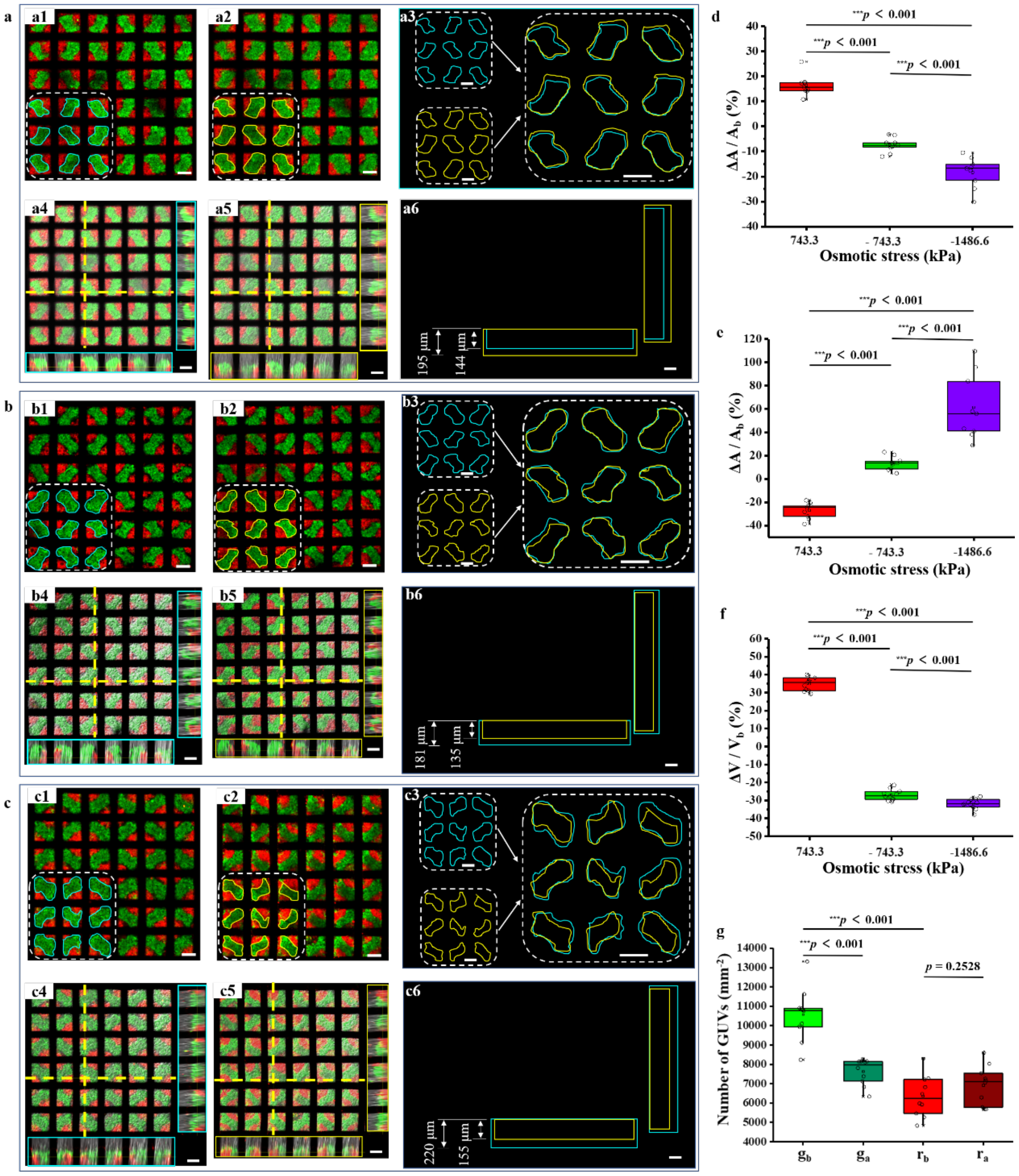

Supplementary Fig. 8 Behaviors of three-component prototissues under different osmotic stress conditions. Behaviors of prototissues under hypotonic condition ($\Delta\Pi$ = 743.3 kPa) **(a)** with pure water solution, hypertonic conditions with 600 mM ($\Delta\Pi$ = -743.3 kPa) **(b)** and 900 mM ($\Delta\Pi$ = -1486.6 kPa) **(c)** glucose solutions. Fluorescence images of the prototissues under isotonic (**a1**, **a4**, **b1**, **b4**, **c1** and **c4**) and under osmotic stress conditions for 30 minutes (**a2**, **a5**, **b2**, **b5**, **c2** and **c5**). The cyan and yellow boxes in **a3**, **b3** and **c3** indicated the areas of the green GUVs populations in the white dashed boxes in **a1, b1, c1** and **a2**, **b2**, **c2**, respectively. The cyan and yellow boxes in **a4, a5**, **b4, b5** and **c4, c5** indicated projected images of prototissues. The cyan and yellow boxes in **a6, b6, c6** indicated the tissue height at isotonic and osmotic stress conditions, respectively. Box plots of the percentage of area variation of the green **(d)** and red **(e)** GUVs populations under different osmotic stresses. $\Delta A/A_b = (A_a\text{-}A_b)/A_b \times 100\%$, where A_b and A_a represented the areas of the green (or red GUVs) populations at isotonic and osmotic conditions, respectively. Data are presented as mean values ± SD (n = 9). Statistical

analyses were carried out by unpaired two-tailed student's t-test (***$p < 0.001$). Source data are provided as a Source Data file. **(f)** Box plots of the percentage of volume variations of the prototissues under different osmotic stress. $\Delta V/V_b = (V_a - V_b)/V_b \times 100\%$, where V_b and V_a represented the volume of the prototissues at isotonic and osmotic conditions, respectively. Data are presented as mean values ± SD (n = 11). Statistical analyses were carried out by unpaired two-tailed student's t-test (***$p < 0.001$). Source data are provided as a Source Data file. **(g)** Box plots of density of the green and red GUVs. g_b, g_a represented the density of green GUVs at isotonic and hypotonic conditions ($\Delta\Pi$ = 743.3 kPa), respectively. r_b, r_a represented the red GUVs at isotonic and hypotonic condition ($\Delta\Pi$ = 743.3 kPa), respectively. Data are presented as mean values ± SD (n = 9). Statistical analyses were carried out by unpaired two-tailed student's t-test (***$p < 0.001$). r_b: r_a: $p = 0.2528$. Vertical center line represented the median. Top and bottom bounds of the box plots represented the first and third quartile. The tips of the whiskers represented min and max values. Source data are provided as a Source Data file. $p < 0.05$ was considered statistically significant. The scale bars were 100 μm.

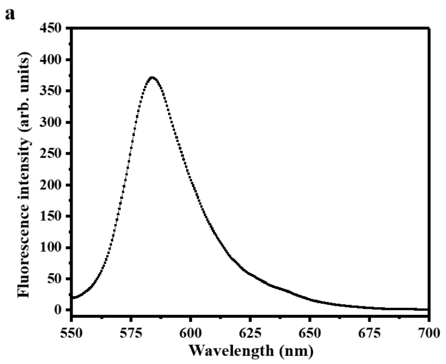

Supplementary Fig. 9 (a) The emission spectrum of resorufin. **(b)** The fluorescence intensity of resorufin in the solution containing free floating GOx-GUVs (1.2×10^6/mL) and HRP-GUVs (4×10^5/mL) as a function of time after the addition of glucose molecules (30 mM) and Amplex Red (0.05 μM) simultaneously (red curve). The fluorescence intensity of Amplex Red (0.05 μM) in the solution without GOx-GUVs, HRP-GUVs, and glucose as a function of time (blue curve), n = 3 independent samples, data are presented as mean values ± SD.

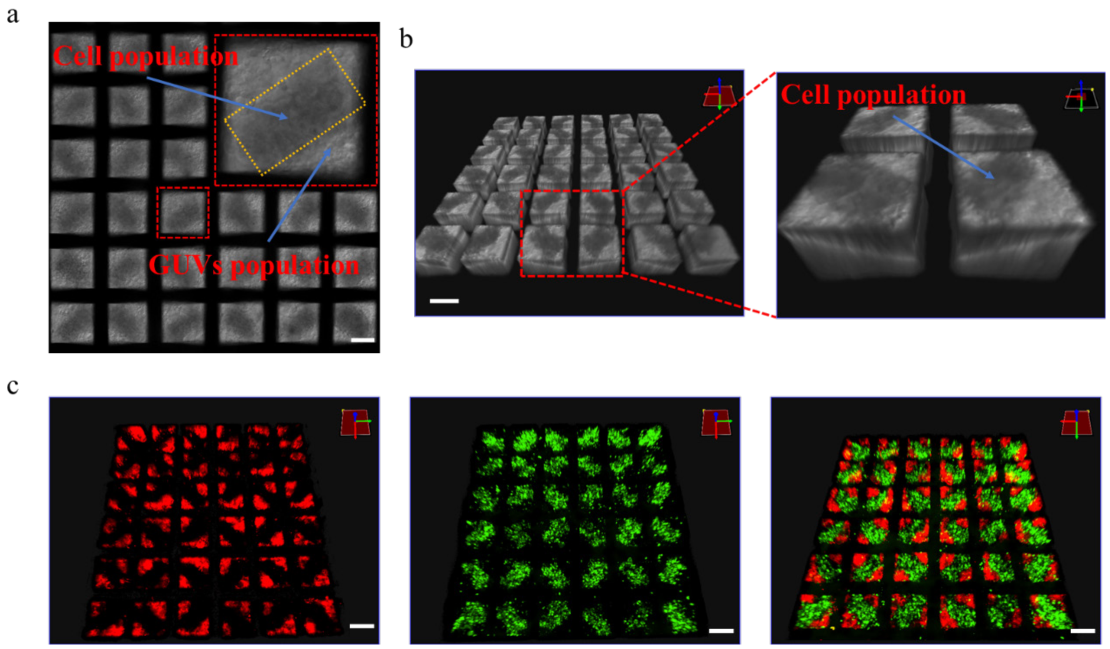

Supplementary Fig. 10 Three-component hybrid prototissues composed of living cells and GUVs. **(a)** Bright field image (from 3 independent samples) of the three-component prototissues. The yellow dashed box indicated the cell population. **(b)** 3D reconstructed bright images of the prototissue showed the relative positions of GVUs populations and living cell populations. The black regions indicated the cell populations and the gray regions indicated the GUVs populations. **(c)** 3D reconstructed confocal fluorescence images of the prototissues of rGVUs populations (left), cell populations (middle) and their merged image (right). The scale bars were 100 μm.

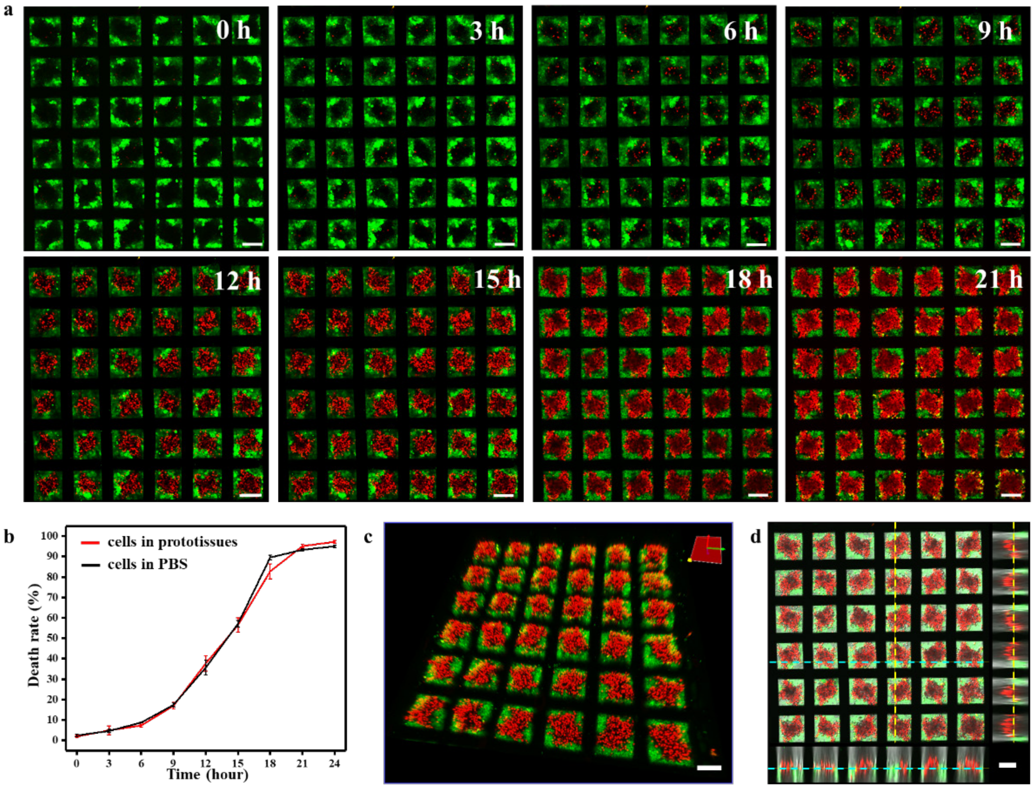

Supplementary Fig. 11 Viability of the glioma cells in the hybrid prototissues over time. **(a)** Fluorescence images (from 3 independent samples) of the hybrid prototissues over time, where red fluorescence and green fluorescence dots indicated the dead cells labelled by propidium iodide (5 μM) and green GUVs populations in PBS solution, respectively. **(b)** The death rates of the glioma cells in the hybrid prototissues and the free glioma cells in PBS solution as a function of time, data are presented as mean values ± SD, n = 3. **(c)** A 3D fluorescence image of the dead cells (red fluorescence) and green GUVs populations in the prototissues in PBS solution for 24 hours. (**d**) A confocal fluorescence image (from 3 independent samples) with projected images of the hybrid prototissues at 24 hours. The gray, green and red regions indicated the non-labeled GUVs at the bottom layer, green GUVs at the edge of top layer, and the cells in the middle of the top layer. The scale bars were 100 μm.

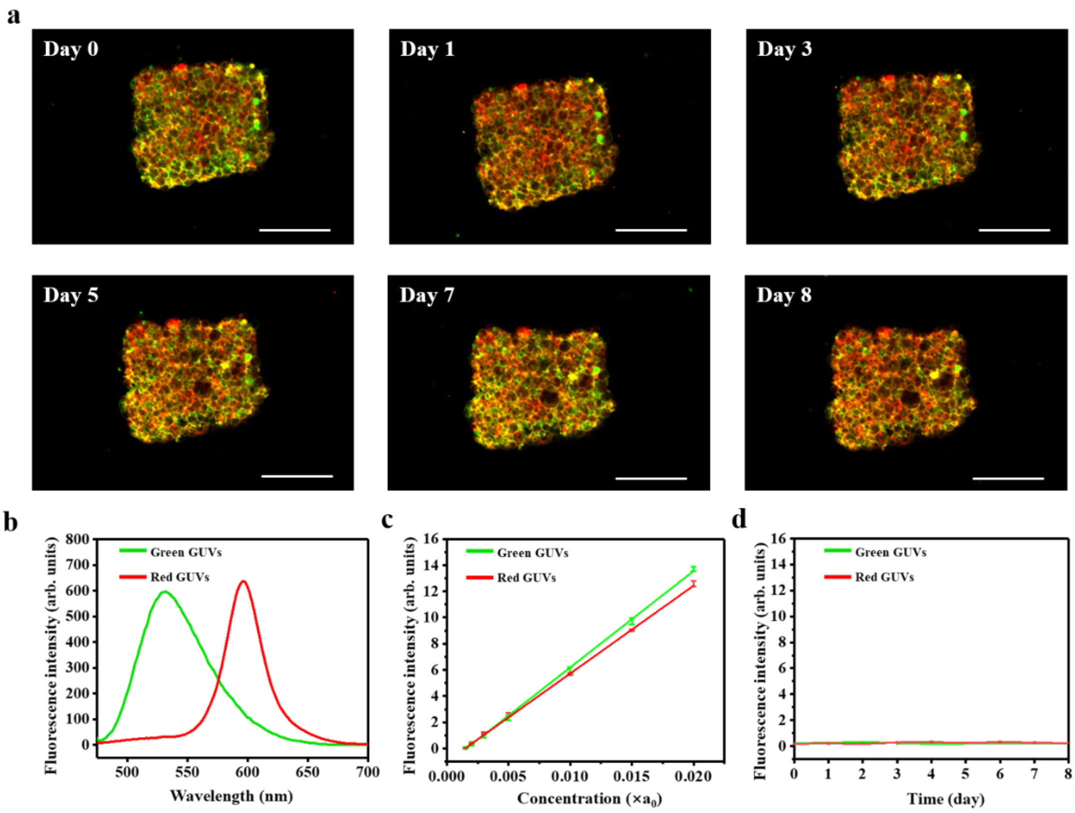

Supplementary Fig. 12 Stability of the detached prototissues. **(a)** The fluorescence images (from 3 independent samples) of a prototissue composed of green and red (1:1) GUVs at different days. The scale bars were 100 μm. **(b)** Fluorescence intensity of the mixed GUVs solution containing NBD-PE labelled GUVs (1×10^6/mL) and Texas red-DHPE labelled GUVs (1×10^6/mL). **(c)** The calibration curves of the GUVs solutions against concentration. a_0 was the concentration of 1×10^6/mL NBD-PE labelled GUVs or 1×10^6/mL Texas red-DHPE labelled GUVs, n = 3 independent samples, data are presented as mean values ± SD. **(d)** Fluorescence intensity of the solution in the petri-dish containing detached same batch prototissues of **(a)** as a function of time, n = 3 independent samples.

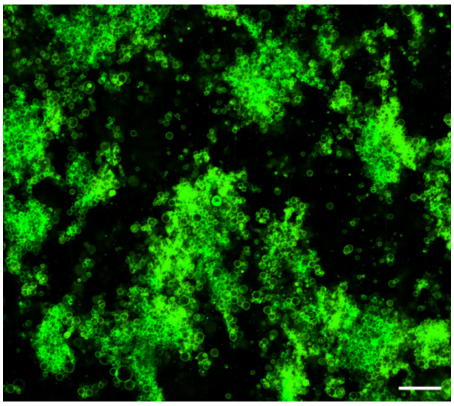

Supplementary Fig. 13 The fluorescence image (from 3 independent samples) of the prototissues composed of green GUVs detached from the NM grids without $CaCl_2$ treatment. The scale bar was 100 μm.

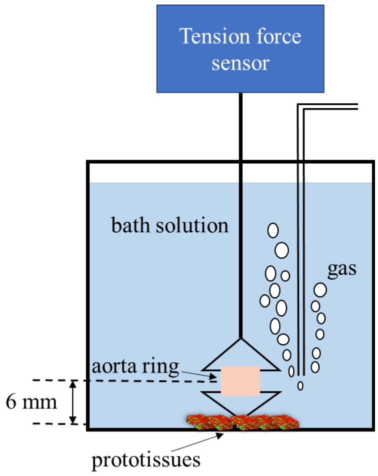

Supplementary Fig. 14 The schematic diagram of the prototissue-induced vasodilation in vitro. The aortic ring was hung on the fixed hooks connected to a force transducer in an organ bath containing 10 mL HEPES solution under physiological O_2 conditions at 37 °C. The prototissues located at the bottom of the bath, which was about 6 mm perpendicularly below the aorta ring.

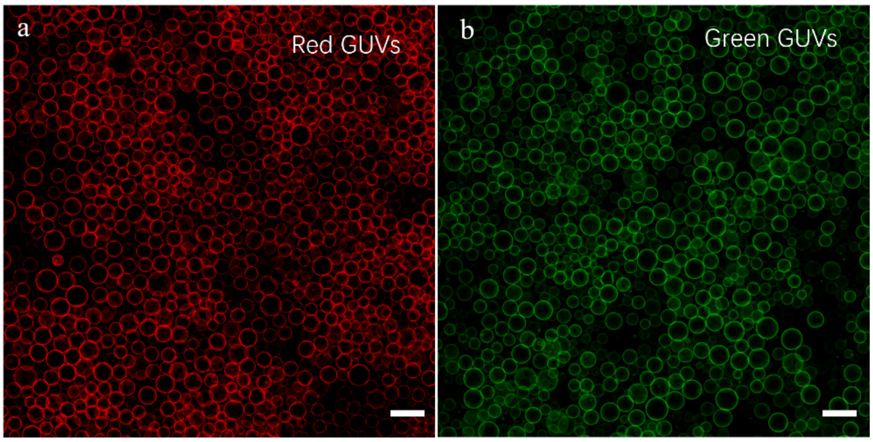

Supplementary Fig. 15 Fluorescence images (from 3 independent samples) of electroformed GUVs. **(a)** GUVs labelled with TR DHPE (rGUVs). **(b)** GUVs labelled with NBD PE (gGUVs). The scale bars were 40 µm.

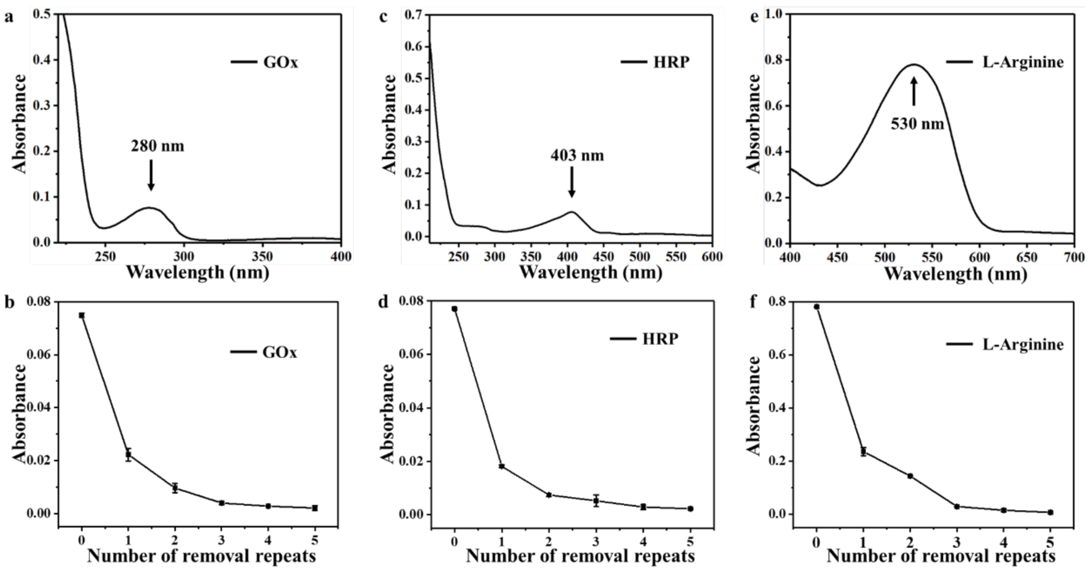

Supplementary Fig. 16 The removal of the free GOx, HRP and L-Arginine in the supernatants. **(a)** UV-Vis absorption spectrum of 30 μg/ml GOx solution. **(b)** Absorbance of supernatants of GOx-GUVs solution at 280 nm as a function of removal repeats. **(c)** UV-Vis absorption spectrum of 20 μg/mL HRP solution. **(d)** Absorbance of supernatants of HRP-GUVs solution at 403 nm as a function of removal repeats. **(e)** UV-Vis absorption spectrum of 3mL 20 mM L-Arginine treated by 3.5 mL Sakaguchi's reagent[1] after 15 minutes. Sakaguchi's reagent contained 2.15 g/L NaOH solution, 42.86 g/L α-naphthol and 2.15 μL/L 2,3-butanedione. **(f)** Absorbance of supernatants of Arginine-GUVs solution at 530 nm as a function of removal repeats. Data are presented as mean values ± SD, n = 3.

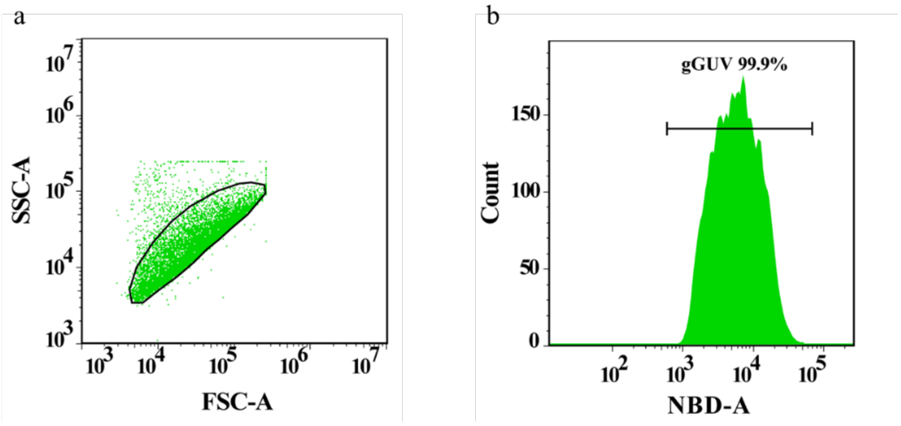

Supplementary Fig. 17 Flow cytometry analysis (from 3 independent samples) was used to calculate the concentration of GUVs. (a) Two-dimensional (2D) dot plots of forward light scattered area (FSC-A) versus side light scattered area (SSC-A) for gGUVs. Total number of particles counted, 10,000. (b) NBD-A counts for populations of gGUVs and corresponding histograms.

Referance

1. Goldschmidt, M. C. & Lockhart, B. M. Simplified Rapid Procedure for Determination of Agmatine and Other Guanidino-Containing Compounds. *Anal. Chem.* **43**, 1475 (1971).
