# Supplementary material for: High-throughput production of functional prototissues capable of producing NO for vasodilation
Source: Nat Commun. 2022 Apr 20;13:2148. doi: 10.1038/s41467-022-29571-3 (PMC9021269; doi:10.1038/s41467-022-29571-3)
Supplement: Supplementary file 3 — Reporting Summary [file 41467_2022_29571_MOESM3_ESM.pdf]

Corresponding author(s): Xiaojun Han

Last updated by author(s): Feb 24, 2022

Reporting Summary

Nature Portfolio wishes to improve the reproducibility of the work that we publish. This form provides structure for consistency and transparency in reporting. For further information on Nature Portfolio policies, see our Editorial Policies and the Editorial Policy Checklist.

Statistics

For all statistical analyses, confirm that the following items are present in the figure legend, table legend, main text, or Methods section.

n/a	Confirmed	
☐	☒	The exact sample size (n) for each experimental group/condition, given as a discrete number and unit of measurement
☐	☒	A statement on whether measurements were taken from distinct samples or whether the same sample was measured repeatedly
☐	☒	The statistical test(s) used AND whether they are one- or two-sided<br>*Only common tests should be described solely by name; describe more complex techniques in the Methods section.*
☒	☐	A description of all covariates tested
☐	☒	A description of any assumptions or corrections, such as tests of normality and adjustment for multiple comparisons
☐	☒	A full description of the statistical parameters including central tendency (e.g. means) or other basic estimates (e.g. regression coefficient) AND variation (e.g. standard deviation) or associated estimates of uncertainty (e.g. confidence intervals)
☐	☒	For null hypothesis testing, the test statistic (e.g. F, t, r) with confidence intervals, effect sizes, degrees of freedom and P value noted<br>*Give P values as exact values whenever suitable.*
☒	☐	For Bayesian analysis, information on the choice of priors and Markov chain Monte Carlo settings
☒	☐	For hierarchical and complex designs, identification of the appropriate level for tests and full reporting of outcomes
☒	☐	Estimates of effect sizes (e.g. Cohen's d, Pearson's r), indicating how they were calculated

Our web collection on statistics for biologists contains articles on many of the points above.

Software and code

Policy information about availability of computer code

Data collection	Fluorescence microscope images data were collected using fluorescence microscope (Olympus IX73, Japan), laser confocal microscope (Olympus FV 3000, Japan) and upright fluorescence microscope (Nikon 80i, Japan). The magnetic field was simulated using COMSOL Multiphysics 5.4 software. The tension force was detected by tension sensor in a constant temperature perfusion system for isolated tissues and organs (TECHMAN HV1403, China). The concentration of giant unilamellar vesicles was estimated using flow cytometry (BD FACSAriaIII flow cytometer, USA).
Data analysis	Fluorescence microscope images were analyzed by CellSens 2.1 and ImageJ 1.8.0. Data process was conducted using Origin 9.0 and Office 2019.

For manuscripts utilizing custom algorithms or software that are central to the research but not yet described in published literature, software must be made available to editors and reviewers. We strongly encourage code deposition in a community repository (e.g. GitHub). See the Nature Portfolio guidelines for submitting code & software for further information.

Data

Policy information about availability of data

All manuscripts must include a data availability statement. This statement should provide the following information, where applicable:

- Accession codes, unique identifiers, or web links for publicly available datasets
- A description of any restrictions on data availability
- For clinical datasets or third party data, please ensure that the statement adheres to our policy

All data that support the findings of this study are available within the paper, its supplementary information, or in the source data.

Field-specific reporting

Please select the one below that is the best fit for your research. If you are not sure, read the appropriate sections before making your selection.

- [x] Life sciences
- [ ] Behavioural & social sciences
- [ ] Ecological, evolutionary & environmental sciences

For a reference copy of the document with all sections, see nature.com/documents/nr-reporting-summary-flat.pdf

Life sciences study design

All studies must disclose on these points even when the disclosure is negative.

Sample size	Every experiments consisted of at least 3 independent samples. Sample size for each experiment was reported in the figure legends. For the signal communication and osmotic stress experiments, the sample sizes were determined according to the previous studies (doi.org/10.1038/s41467-019-14141-x). For the thoracic aorta ring tests, the samples were 5 vascular rings, which was commonly exploited by researchers in the field (doi.org/10.1038/ncomms14807).
Data exclusions	No such exclusion in this paper.
Replication	All experiments were independently replicated at least three times. The reproducible results were always obtained.
Randomization	Wistar rats aged from 10-12 weeks were randomly selected to obtain thoracic aorta rings. Thoracic aorta rings were randomized into groups.
Blinding	No blinding of experiments was necessary.

Reporting for specific materials, systems and methods

We require information from authors about some types of materials, experimental systems and methods used in many studies. Here, indicate whether each material, system or method listed is relevant to your study. If you are not sure if a list item applies to your research, read the appropriate section before selecting a response.

Materials & experimental systems

n/a	Involved in the study	
x		Antibodies
	x	Eukaryotic cell lines
x		Palaeontology and archaeology
	x	Animals and other organisms
x		Human research participants
x		Clinical data
x		Dual use research of concern

Methods

n/a	Involved in the study	
x		ChIP-seq
	x	Flow cytometry
x		MRI-based neuroimaging

Eukaryotic cell lines

Policy information about cell lines

Cell line source(s)	C6 glioma cells were obtained from Procell Life Science&Technology (CL-0047, China).
Authentication	The cell line was authenticated by PCR assays.
Mycoplasma contamination	Mycoplasma contamination was not tested. C6 glioma cells were used to investigate the change in the amount of nitric oxide inside them.
Commonly misidentified lines (See ICLAC register)	No commonly misidentified lines were used in this study.

Animals and other organisms

Policy information about studies involving animals; ARRIVE guidelines recommended for reporting animal research

Laboratory animals	Male Wistar rats, aged 10-12 weeks, were obtained from Animal Laboratory Center of The First Affiliated Hospital of Harbin Medical University.
Wild animals	No wild animals were used in the study.
Field-collected samples	No field collected samples were used in the study.

Ethics oversight	We confirmed that ethical approval from the Experimental Animal Ethics Committee of Harbin Institute of Technology (IACUC-2021012) was obtained prior to the study.

Note that full information on the approval of the study protocol must also be provided in the manuscript.

Flow Cytometry

Plots

Confirm that:

- [x] The axis labels state the marker and fluorochrome used (e.g. CD4-FITC).
- [x] The axis scales are clearly visible. Include numbers along axes only for bottom left plot of group (a 'group' is an analysis of identical markers).
- [x] All plots are contour plots with outliers or pseudocolor plots.
- [x] A numerical value for number of cells or percentage (with statistics) is provided.

Methodology

Sample preparation	All analyzed samples are giant unilamellar vesicles (GUVs) labeled with NBD PE, which are not living organisms. GUVs were prepared using the method as stated in the Methods section.
Instrument	BD LSRFortessa Flow cytometry
Software	FlowJo 7.6.1
Cell population abundance	Total number of particles counted, 10000
Gating strategy	Forward and side scatter gating

- [x] Tick this box to confirm that a figure exemplifying the gating strategy is provided in the Supplementary Information.
